# Malignant atrophic papulosis treated with eculizumab and hirudin: a fatal case report and literature review

**DOI:** 10.3389/fcvm.2024.1347587

**Published:** 2024-03-28

**Authors:** Linna Yu, Yun Wang, Xiaodan Tang, Xueru Zhao, Zhengji Song

**Affiliations:** ^1^ Department of Gastroenterology, The First People’s Hospital of Yunnan Province, Kunming, China; ^2^ The Affiliated Hospital of Kunming University of Science and Technology, Kunming, Yunnan, China

**Keywords:** malignant atrophic papulosis, complement, interferon, vascular disease, eculizumab, treprostinil

## Abstract

**Background:**

Malignant atrophic papulosis (MAP) is a rare obliterative vasculopathy whose etiology and pathophysiological mechanisms remain unknown, and the treatment is still empirical. It can involve multiple systems, especially the gastrointestinal tract and central nervous system, and has a poor prognosis.

**Case presentation:**

A 20-year-old Chinese male appeared to have Widespread atrophic papules and plaques, intermittent abdominal pain, recurrent bowel perforation, and psoas abscess. The clinical diagnosis of MAP was supported by skin biopsy. He was then treated with anticoagulants, antiplatelets, glucocorticoids, and immunosuppressants and started on eculizumab and hirudin after the first surgical interventions. Despite the aggressive immunosuppression, anticoagulant, antiplatelet, humanized monoclonal antibodies, and surgery therapy, he died five months after presentation.

**Conclusions:**

MAP is an extremely rare obliterative vasculopathy manifesting as benign cutaneous involvement or potentially malignant systemic involvement. MAP patients who exhibit any abdominal symptoms should undergo laparoscopy and evaluation in time and start on eculizumab and treprostinil as soon as possible, as the combination of them is presently the most effective treatment option for gastrointestinal MAP and hopefully reduce mortality.

## Introduction

Malignant atrophic papulosis (MAP) is an extremely rare obliterative vasculopathy manifesting as potentially malignant systemic involvement (1). Notably, MAP has a poor prognosis and is almost universally fatal (2, 3). The most common cause of death was recurrent bowel perforation, followed by neurologic events and cardiac outcomes (4–6). Although most individuals develop symptoms between the ages of 20 and 50, there are also case reports outside this age range (7, 8). MAP is most commonly diagnosed in Caucasians and is rarely reported in Asians. This case report provides additional evidence that MAP can affect people of all ages and races, but few family events have been reported. The exact underlying mechanism of MAP remains unclear, and effective treatments are still being developed. Fewer than 200 patients have been reported in the medical literature, and less than 50 patients worldwide are known to be alive (9). However, due to the rarity of the disease, many individuals may be undiagnosed or diagnosed only at autopsy (7).

The cutaneous manifestation typical of MAP is porcelain-white atrophic lesions with a telangiectatic rim (10). The lesions mainly occur on the trunk and are distributed centripetally, ranging from a few to hundreds, recurring in batches, and only a few rashes appear each time, which are scattered distribution and rarely coalesce (11). These lesions are usually small in number and asymptomatic, often absent from the face and hands, and thus may go unnoticed (1). Patients' awareness of the lesions ranges from a few days to nearly 30 years (4). It is worth emphasizing that MAP can also involve other organs, especially the gastrointestinal tract, and has a poor prognosis.

Here, we report a fatal case of malignant atrophic papulosis with widespread atrophic papules, recurrent bowel perforation, and psoas abscess. We report this case to highlight the importance of early diagnosis and treatment, to make physicians deeply understand the clinical manifestations of the disease and its responses to various medications, and to provide the basis for developing individualized treatment plans.

This patient diagnosis and treatment are outlined in the timeline below (Supplementary Figure S1).

## Case

A 20-year-old Chinese male presented to the First People's Hospital of Yunnan Province in July 2022 with a 1-year history of widespread porcelain-white papules and plaques and a 2-month history of intermittent abdominal pain.

Symptoms began at the age of 19 years with painless skin lesions on his abdomen, and subsequently, the new painless skin lesions gradually developed on his chest, back, and extremities. Treatment with topical broad-spectrum antibiotics, corticosteroids, or skin moisturizers was initiated without benefit. The following year, at the age of 20 years, the patient developed abdominal pain or discomfort that was relieved after defecation rest, but new rashes appeared intermittently in small quantities. Two months later, the patient developed new-onset paroxysmal, crampy abdominal pain with associated nausea or vomiting and visited the First People's Hospital of Yunnan Province for further assessment. Contrast-enhanced abdominal computed tomography revealed enteritis, colitis, intestinal obstruction, and a small amount of pelvic fluid (Supplementary Figure S2).

The patient was otherwise in good health without any other significant medical history, and he is not currently taking any medications. No chronic diseases, no alcohol use, no family history, no herbal agents, or no suspected drug use were reported.

## Diagnostic testing and assessment

**Figure 1:**
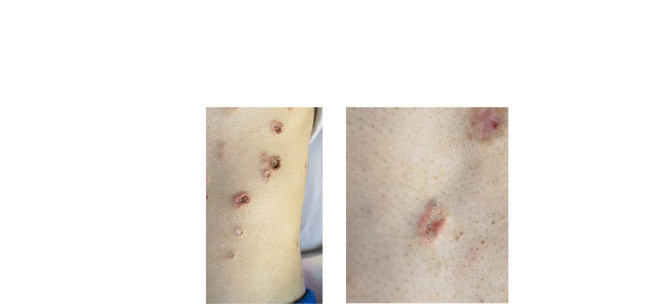
Malignant atrophic papulosis. (**A**) The porcelain-white, irregularly shaped atrophic papules with erythematous rims of telangiectasia on the abdomen, some of them developed scabs in the center. On August 23, the patient underwent exploratory laparotomy due to the first intestinal perforation, leading to the scar. (**B**) Close-up view of atrophic papules.

The skin examination at the First People's Hospital of Yunnan Province showed miliary to soybean-sized atrophic papules widespread on the trunk and extremities of the patient except for the face, neck, palms, and soles of feet. These papules with porcelain white centers and clear boundaries were irregular in shape and developed black scabs in some centers (Figure 1). At the same time, a distinct rim of intense erythema with telangiectasia was characterized by all lesions, and adjacent lesions formed larger atrophic plaques in several areas, including the abdomen, back, and proximal thighs. In addition, his skin examination revealed several healed scars and a small proportion of active inflammatory lesions did not evolve into atrophic porcelain-white macules. Physical examination was highly suggestive of MAP.

**Figure 2:**
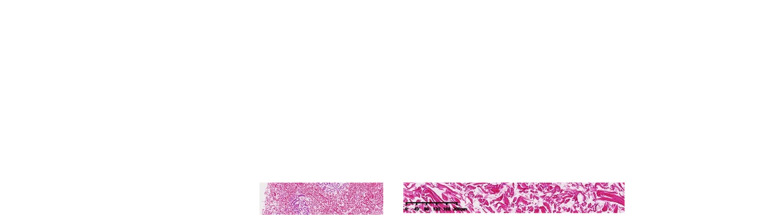
Dermatopathology of malignant atrophic papulosis. (**A**) Local epidermal atrophy with mild keratinization, local tissue necrosis with infiltration of inflammatory cells, and increased dermal mucin. (**B**) Fibrous occlusion of the arteriolar lumen and mild fibrous thickening of the intima of venules, and lymphocytes and individual plasma cells can be seen gathering and infiltrating around the above blood vessels. (**A** and **B**, Hematoxylin-eosin stain; original magnification: **A**, ×40; **B**, ×200).

The following laboratory studies were significant for diagnosis: fibrinogen 5.46 g/L (reference range: 2–4 g/L), fibrinogen degradation products 12.15 ug/ml (reference range: 0-5 ug/ml), D-dimer 4.10 ug/ml (reference range: 0–0.05 ug/ml), erythrocyte sedimentation rate 43 mm/h (reference range: 0–15 mm/h), procalcitonin 0.440 ng/ml (reference range: 0–0.052 ng/ml), C-reactive protein 52.30 mg/L (reference range: 0–3.0 mg/L), hypersensitive CRP 98.92 mg/L (reference range: 0–10 mg/L), red blood cell count 4.13 × 10^12^/L (reference range: 4.3–5.8 × 10^12^/L), hemoglobin level 114 g/L (reference range: 130–175 g/L), fecal occult blood test (positive); white blood cell, platelet count and other biochemical, virological and immunological blood test results were within the normal limits. Especially anti-nuclear antibodies, anti-DNA antibodies, anti-Sm antibodies, anti-Scl-70 antibodies, anti-cardiolipin antibodies, lupus anticoagulant, anti-SSA/Ro antibodies, anti-SSB/La antibodies, anti-neutrophil cytoplasmic antibodies were within the normal limits. Skin biopsy revealed fibrous occlusion of the arteriolar lumen and mild fibrous thickening of the intima of venules, local epidermal atrophy with mild keratinization, local tissue necrosis with infiltration of inflammatory cells, and increased dermal mucin. It supported the clinical diagnosis of MAP (Figure 2). Contrast-enhanced abdominal computed tomography revealed that MAP might involve the gastrointestinal tract. The electrocardiogram showed sinus rhythm and was within the normal limits.

The differential diagnosis of MAP includes lymphomatoid papulosis, pityriasis lichenoides et varioliformis acuta, segmental hyalinizing vasculitis, allergic cutaneous vasculitis, antiphospholipid syndrome, and systemic lupus erythematosus. Lymphomatoid papulosis, pityriasis lichenoides et varioliformis acuta, segmental hyalinizing vasculitis, and allergic cutaneous vasculitis can be distinguished by skin biopsy. However, MAP is frequently indistinguishable from systemic lupus erythematosus by histopathologic and immunopathologic, but systemic lupus erythematosus often presents with typical butterfly-shaped erythema on the face. The detection of anticardiolipin antibodies can help distinguish MAP from antiphospholipid syndrome.

## Treatment

The patient received fasting, gastrointestinal decompression, nutritional support, and ceftriaxone. He was temporarily not receiving glucocorticoids, anticoagulation, and antiplatelet treatment before the evaluation of laparoscopy due to concerns about the increased risks of gastrointestinal bleeding, perforation, and infection. However, the patient refused laparoscopy and requested to be referred to another hospital for re-evaluation on July 13. The diagnosis was consistent with that of our hospital, and he started to receive anticoagulants, antiplatelets, glucocorticoids, immunosuppressants, and proton pump inhibitors. Although the symptoms were alleviated, the patient continued to experience intermittent abdominal pain that was accompanied by worsening skin lesions. During this time, the patient was very uncomfortable and wasting away due to persistent and progressive gastrointestinal disease.

On August 18, the patient returned with recurrent abdominal pain that was exacerbated due to the onset of severe pain for nearly a week. He received Low-molecular-weight heparin calcium injection (4,100 IU ih qd), aspirin (100 mg po qd), dipyridamole (50 mg po tid), meprednisone (40 mg iv gtt qd), thalidomide (50 mg po tid), pantoprazole (40 mg iv gtt qd), moxifloxacin (0.4 g iv gtt qd) and nutritional support. On August 23, his abdominal pain worsened. Computed tomography of the abdomen revealed enteritis, colitis, gastrointestinal perforation, and peritonitis (Supplementary Figure S3). He underwent an exploratory laparotomy immediately, and it was found that the transverse colon adhered to the duodenum, gas was expelled, and there was a large number of pus on the surface. The scattered purulent lesions were found on the serosa of the remaining colon and small intestine, which were similar in appearance to the lesions on his skin. Some lesions were black in the center. Small intestinal perforation was discovered in the lesions 50 cm and 80 cm away from the ileocecal. Eventually, he underwent a colostomy and revision of intestinal perforation (Supplementary Figure S4).

After the operation, he received meropenem (1 g q8h), proton pump inhibitors, bowel rest, and parenteral nutrition. The above anticoagulant, antiplatelet, glucocorticoid, and immunosuppressant were stopped for one week. After assessing that there was no infection in the patient and confirming that he had been vaccinated against meningococcal meningitis, the weekly intravenous administration of eculizumab 600 mg was added to his ongoing drug regimens on September 5. Although he reported a slight improvement in abdominal pain, there was a deterioration in clinical conditions. Laboratory tests showed that fecal occult blood test (positive), APTT 46.5 s (reference range: 28.0–43.5 s), TT 29.7 s (reference range: 11.0–15.0 s), suggesting a high risk for anticoagulation. Therefore, the traditional anticoagulant and antiplatelet were replaced with hirudin (0.5 g po qd) under the guidance of hematology experts On September 14. The patient reported improvement in abdominal pain and was discharged from the hospital on September 19, although there were no significant changes in skin lesions, and abdominal symptoms kept persisting. He kept receiving eculizumab (600 mg iv q1w), prednisone (30 mg po qd), and thalidomide (50 mg po qn) after discharge.

On September 26, the patient returned due to new-onset soreness and pain in the right lumbar region. Contrast-enhanced abdominal computed tomography revealed the right psoas abscess extending to the perinephric space (Supplementary Figure S5), and eculizumab was thereby discontinued. When placing a jejunal feeding tube, multiple ulcers in the esophagus and stomach of the patient were discovered (Supplementary Figure S6). However, the glucocorticoid, anticoagulant, and other drug treatments were not discontinued. On October 11, the patient underwent another laparotomy for acute severe abdominal pain. In addition to 2 new-onset proximal small bowel perforations, an extensive retroperitoneal abscess was found. After surgery, the patient was in critical condition and transferred to the intensive care unit for further treatment. After surgery, the patient was in critical condition and transferred to the intensive care unit for further treatment, and subsequently, his clinical condition deteriorated rapidly. In compliance with his family's requirements, the patient was discharged on October 20.

The patient died in November 2022, presumably of sepsis and multiorgan failure, and no autopsy was performed. It was less than five months from his first visit to the First People's Hospital of Yunnan Province with abdominal pain for evaluation to death.

## Discussion

Malignant atrophic papulosis (MAP), also known as Degos disease, is an extremely rare disease that is characterized by its unique skin presentation. Some patients who presented with only cutaneous symptoms at first may develop systemic symptoms several years later (7). The gastrointestinal tract is most often affected in MAP, followed by the central nervous system and cardiovascular system (4, 12–14). According to reports, the lower rather than the upper gastrointestinal tract is predominantly affected in MAP (11). However, the patient developed extensive lesions from the esophagus to the colon, which provides additional evidence that MAP could affect any part of the gastrointestinal tract. It has been reported that gastrointestinal lesions frequently lead to intestinal perforation. However, it is worth noting that the time from the onset of abdominal manifestation to intestinal perforation is variable and unpredictable, and most perforations were discovered by emergency laparotomy or an autopsy (4). However, there is almost always an opportunity for diagnosis and intervention before such catastrophic outcomes occur.

Although no specific laboratory tests and imaging examinations are available for diagnosing MAP, typical skin manifestations and histologic findings are helpful. MAP exhibits a peculiar and distinctive predilection to involve select small arteries and arterioles of the subserosa (15). Laparoscopy will reveal pathognomic lesions on the serosal surface of the intestine. Therefore, laparoscopic evaluation is the most effective way to diagnose gastrointestinal MAP and assess treatment response (4, 6), but it is underutilized. In addition, laparoscopy is considered a more sensitive diagnostic approach than endoscopy. Some research points out that, endoscopy should not be performed before laparoscopy due to increasing the risk of perforation (15). Since the patient has been refusing laparoscopy, the widespread lesions in the gastrointestinal tract were found until laparotomy, and he rapidly died of repeated bowel perforations. Therefore, patients with MAP who exhibit any abdominal symptoms should undergo laparoscopy and evaluation in time to promote diagnosis and intervention, and it hopefully reduces mortality.

Although MAP has been known for about 70 years, the pathogenesis and pathophysiology of the disease remain unclear. It is believed by some researchers to be an autosomal dominant disorder and has a familial inheritance pattern (16, 17). However, the patient had no significant family history. The pathogenesis of MAP is suspected to be related to viral infection, autoimmune dysfunction, dysregulation of interferon-α signaling, and activation of the complement cascade (18, 19). However, the relevant laboratory tests in the patient did not reveal evidence of viral infection or autoimmune disease. In a recent study by Magro et al., MAP was shown to be a syndrome of vascular injury involving deposition of complement component C5-9 complex and high expression of interferon-α (4). Based on current knowledge, we speculated that this patient may have MAP associated with C5b-9 complex deposition and high interferon-α expression.

MAP vascular diseases include thrombotic microangiopathies targeting capillaries and venules and stenotic fibrointimal arteriopathy involving small and medium arteries (20, 21). One possibility for the thrombotic mechanism in MAP is the increased intima-media thickness (IMT), as it is currently seen in other microthrombotic diseases like antiphospholipid syndrome (22). According to recent reports, MAP benefits from antiplatelet treatment (23–27). However, despite the coagulation abnormalities of the patient, the skin lesions did not benefit from the intensive anticoagulant and antiplatelet therapy that also failed to prevent the rapidly progressive deterioration of the gastrointestinal injury, suggesting that thrombosis in MAP may be a secondary event. Traditional anticoagulants and antiplatelet therapies may promote the progression of gastrointestinal damage, and some studies prove that hirudin has minor bleeding side effects and rarely causes toxicity (28). Therefore, we experimentally used hirudin under the guidance of hematology experts. To our knowledge, this is the first patient with MAP to be anticoagulated with hirudin. He achieved transient subjective minor improvement in abdominal symptoms, although the skin reaction was not obvious, which provided new insights into the treatment of MAP. However, the study has certain limitations due to a single sample. Therefore, larger and more comprehensive studies are necessary in the future to confirm the therapeutic effects of Hirudin.

The patient also received glucocorticoids and thalidomide. It is worth emphasizing that he developed intestinal perforations one month later, which occurred again one month after reusing the above medications after the operation, as well as retroperitoneal abscesses. In addition, the skin lesions got worse. This raises the question of whether glucocorticoids and thalidomide contributed to the development of intestinal perforations and the progression of skin lesions. It seems to be possible, given the dose and duration of administration. Indeed, it has been reported that skin lesions deteriorated during immunosuppression with prednisolone, azathioprine, and cyclosporine (29). In another case, the skin lesions benefited from cyclosporine, but the report is limited (30).

Eculizumab is a humanized monoclonal antibody preventing the cleavage of human complement component C5 into its proinflammatory components and has antiapoptotic endothelial cell effects (19, 31). Eculizumab has an immediate effect on improving endothelial cell damage and venous thrombosis in both small and large vessels, and it is considered a salvage treatment for critically ill patients with thrombotic microangiopathy despite being expensive (2, 4, 31). It has been reported that five of the post-perforation patients survived receiving Eculizumab treatment (4). The patient did not start receiving eculizumab until extensive lesions on the intestinal serosa were discovered at laparotomy. Eculizumab was purchased from the manufacturer by the patient's family and used after approval by the hospital. Although the skin lesions had no significant change after the infusion of eculizumab, the patient reported a slight improvement in abdominal pain. However, he experienced bowel perforation again shortly afterward, suggesting that eculizumab may have a salvage therapeutic effect to a certain extent but not sufficient to improve the extensive intestinal lesions that have already developed. In addition, a study has shown that eculizumab has little effect on occlusive fibrointimal artery disease (4). Therefore, early prevention of progressive changes in occlusive non-thrombotic fibrointimal arteries may be the key to preventing catastrophic outcomes.

It has been reported that eculizumab cannot provide sustained remission of gastrointestinal symptoms (32, 33), but the combination of treprostinil and eculizumab is encouraging (33, 34). The mechanism of treprostinil in this condition is unknown, possibly increasing the number of circulating endothelial cells and allowing angiogenesis (9). Treprostinil was not used in the patient due to high costs and insurance challenges. However, although eculizumab and treprostinil have been continued, progressive neurologic, pleural, and pericardial MAP has been reported (4, 35). A recent study has revealed that the obliterative proliferative neointimal arteriopathy and the sclerodermic fibrosing serositis occur independently of complement inhibition, and both of them occurred in patients exhibiting complete blockade of complement on eculizumab (4). Therefore, it has been suggested that enhanced type I interferon signaling may be a key determinant of intimal expansion (36).

In conclusion, the therapies discussed above will undoubtedly change as the disease mechanisms are better understood. Drugs that directly target the interferon pathway and others with anti-interferon activity have now been developed, but there have been no reports on the use of these drugs. No doubt, therapies to prevent and reverse the neointimal hyperplasia associated with malignant atrophic papulosis will also be discovered. Therefore, malignant atrophic papulosis is an increasingly treatable disease, especially when recognized and diagnosed in time.

## Data Availability

The original contributions presented in the study are included in the article/Supplementary Material, further inquiries can be directed to the corresponding author.
