# Supplementary material for: Malignant atrophic papulosis treated with eculizumab and hirudin: a fatal case report and literature review
Source: Front Cardiovasc Med. 2024 Mar 28;11:1347587. doi: 10.3389/fcvm.2024.1347587 (PMC11007069; doi:10.3389/fcvm.2024.1347587)

Supplementary Figure 1. Timeline of patient diagnosis and treatment.

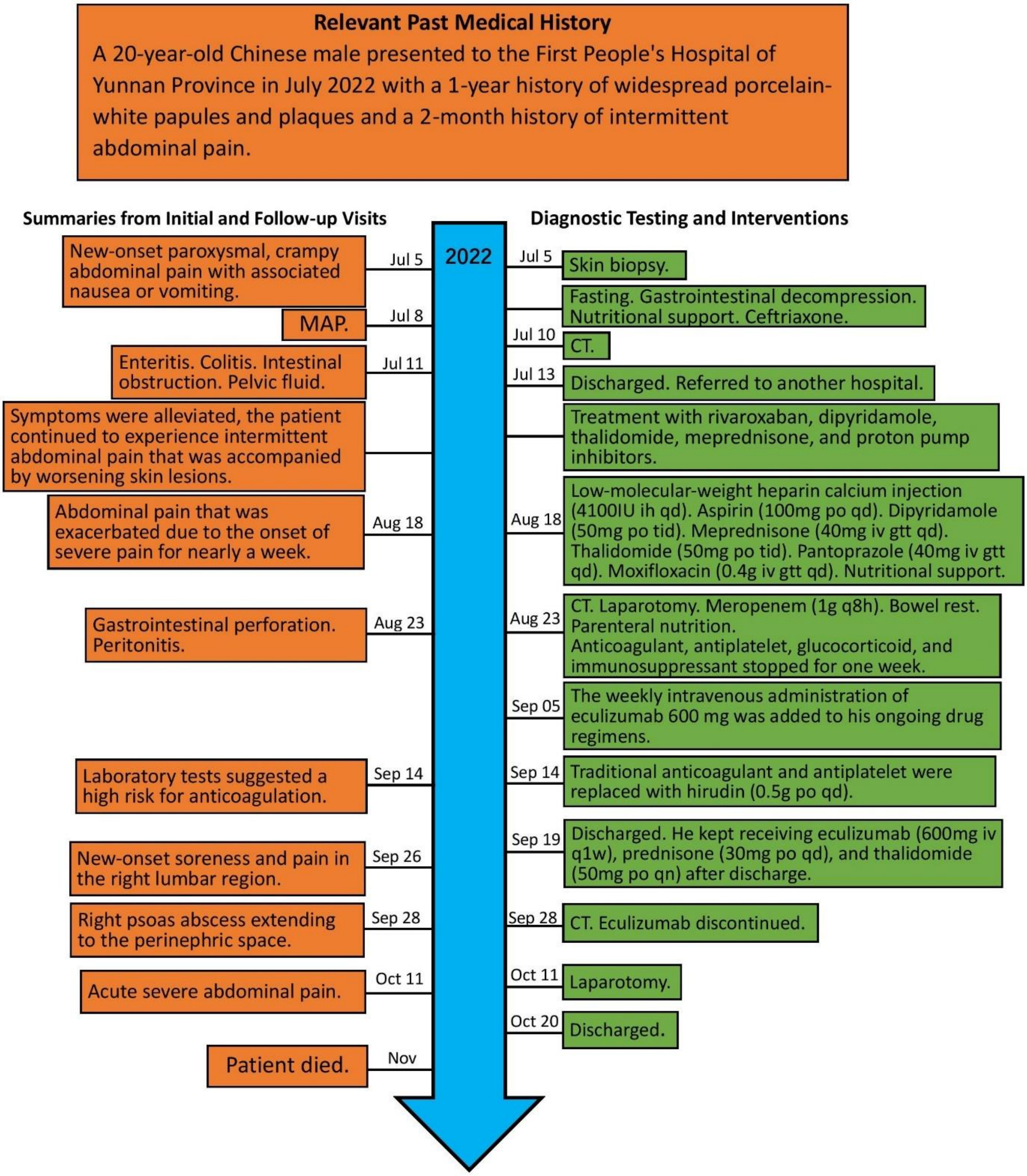

Abbreviations: CT, computed tomography; MAP, malignant atrophic papulosis.

Supplementary Figure 2. Abdominal computed tomography with contrast. **A**, The walls of the small intestine and ascending colon in the right middle of the upper abdomen were thickened, and the serosal surface was not smooth. **B**, Intestinal pneumatosis, effusion, dilatation, and air-fluid level were shown.

A

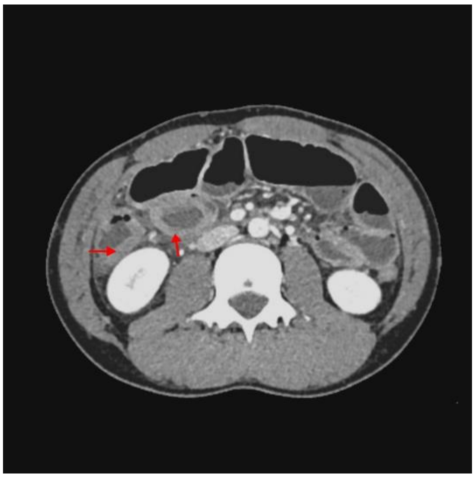

B

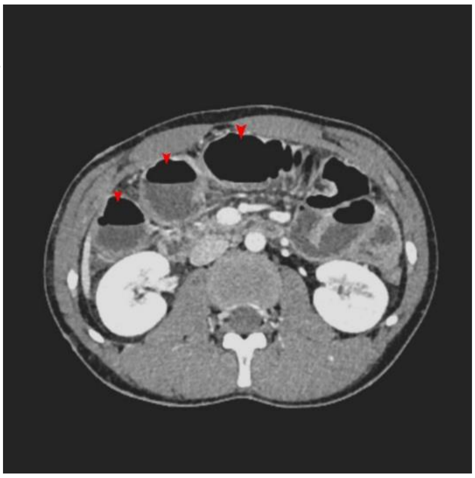

Supplementary Figure 3. Abdominal computed tomography without contrast. **A**, Free gas was scattered in the abdominal cavity. **B**, The focal transverse colon wall was thickened, and patchy gas shadows were shown on the upper edge of the transverse colon.

A

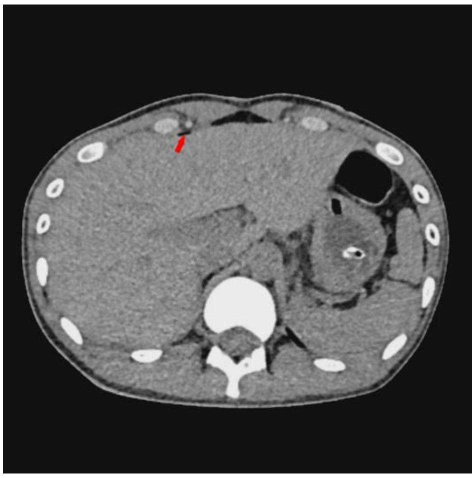

B

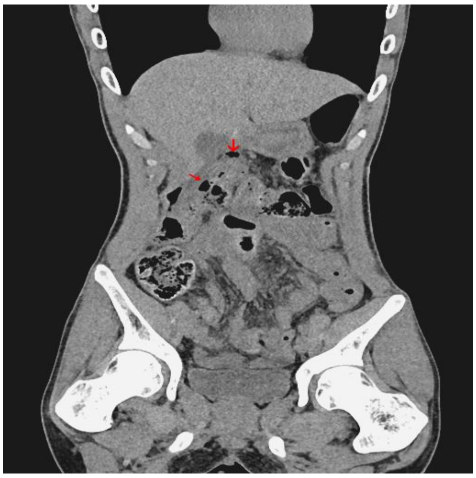

Supplementary Figure 4. Intraoperative findings. **A**, Patchy necrosis and subserosal porcelain white plaque lesions on the small intestine. **B**, Multiple perforations and subserosal porcelain white plaque lesions.

A **B**

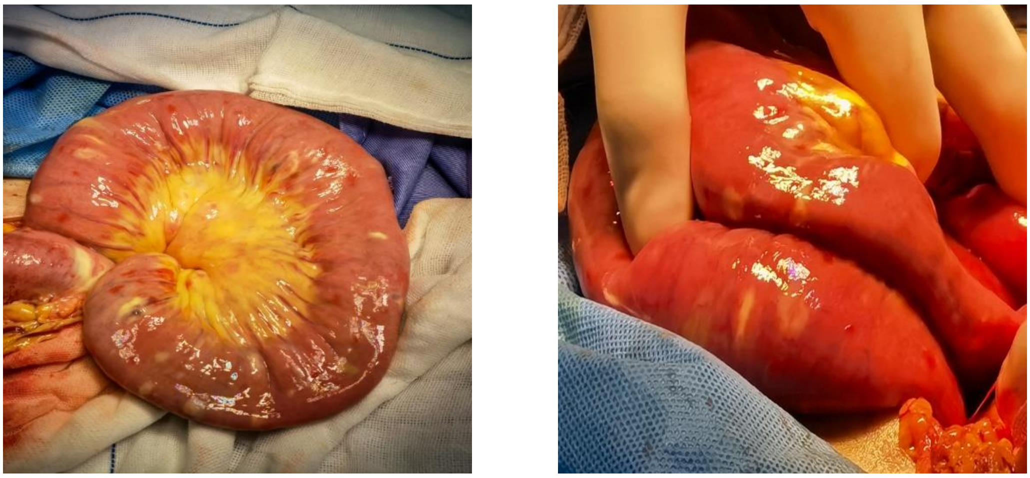

Supplementary Figure 5. Abdominal computed tomography with contrast showed the right psoas abscess extended to the perinephric space (**A-B**) and an ostomy shadow in the middle of the abdomen (**B**).

A **B**

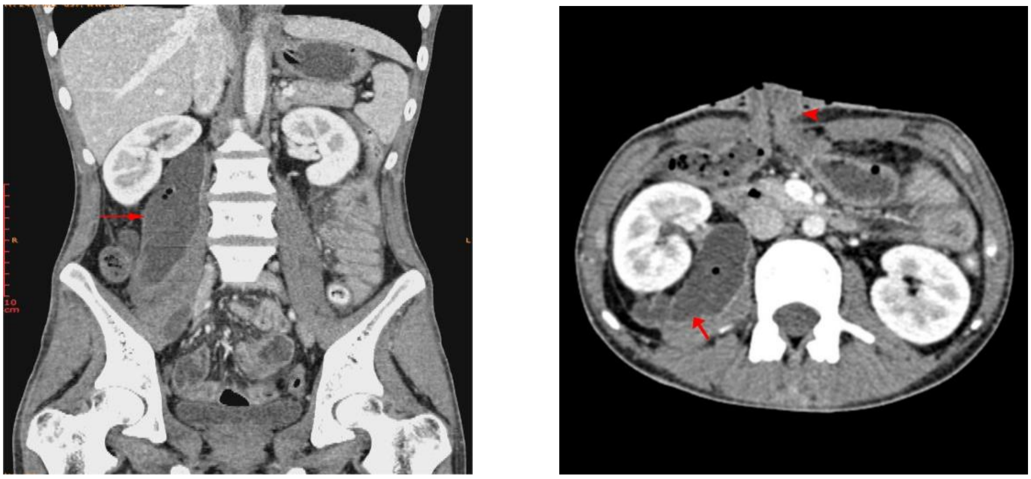

Supplementary Figure 6. Esophagogastroduodenoscopy showed multiple ulcerations with surrounding edema in the stomach (**A**) and esophagus (**B**).

A **B**

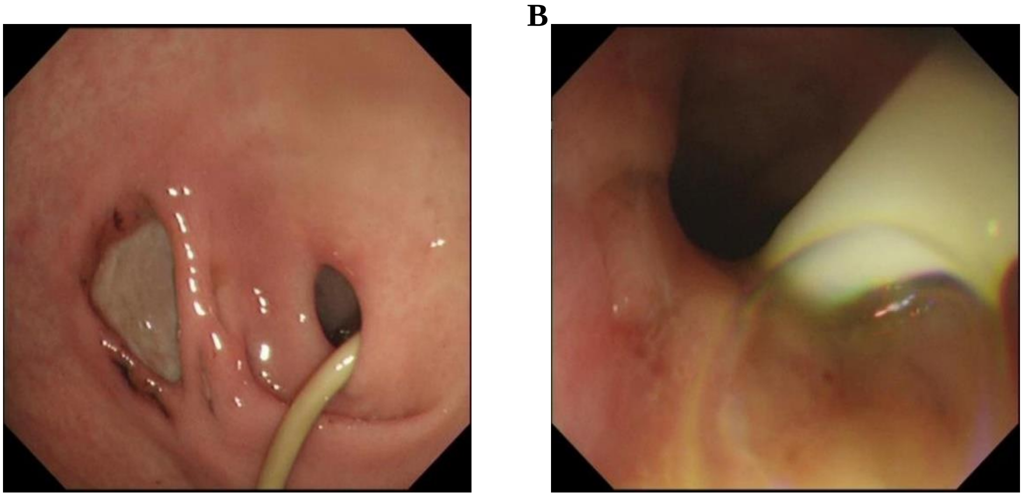

Supplement: Supplementary file 1 [file Datasheet1.pdf]
